# scPOEM: robust co-embedding of peaks and genes revealing peak–gene regulation

**DOI:** 10.1093/bioinformatics/btaf483

**Published:** 2025-09-01

**Authors:** Yan Zhong, Yuntong Hou, Yongjian Yang, Xinyue Zheng, James J Cai

**Affiliations:** KLATASDS-MOE, School of Statistics, East China Normal University, Shanghai, 200062, China; KLATASDS-MOE, School of Statistics, East China Normal University, Shanghai, 200062, China; Department of Electrical and Computer Engineering, Texas A&M University, College Station, TX 77843, United States; KLATASDS-MOE, School of Statistics, East China Normal University, Shanghai, 200062, China; Department of Electrical and Computer Engineering, Texas A&M University, College Station, TX 77843, United States; Department of Veterinary Integrative Biosciences, Texas A&M University, College Station, TX 77843, United States; Interdisciplinary Program of Genetics, Texas A&M University, College Station, TX 77843, United States

## Abstract

**Motivation:**

Identifying regulatory elements in various chromosomal regions that influence gene expression is a fundamental challenge in epigenomics, with profound implications for understanding gene regulation and disease mechanisms. The advent of paired single-cell RNA sequencing and single-cell ATAC sequencing has created unprecedented opportunities to address this challenge by enabling simultaneous profiling of gene expression and chromatin accessibility at single-cell resolution. However, the inherent signals between them are weak due to the highly sparse and noisy nature of data.

**Results:**

This article proposes single-cell meta-Path based Omics Embedding (scPOEM), a novel embedding method that jointly projects chromatin accessibility peaks and expressed genes into a shared low-dimensional space. By integrating the relationships among peak–peak, peak–gene, and gene–gene interactions, scPOEM assigns closer representations in the embedding space to related peak–gene pairs. Our experiments demonstrate that scPOEM generates stable representations of peaks and genes, outperforms existing methods in recovering biologically meaningful peak–gene regulatory relationships and enables new insights in subgroup and differential analysis of gene regulation. These results highlight its potential to uncover gene regulatory mechanisms and enhance the understanding of transcriptional regulation at single-cell resolution.

**Availability and implementation:**

The source code of scPOEM is available at https://github.com/Houyt23/scPOEM. The datasets can be obtained from the 10× Genomics (https://www.10xgenomics.com/datasets/pbmc-from-a-healthy-donor-granulocytes-removed-through-cell-sorting-10-k-1-standard-1-0-0) and GEO database under access codes GSE194122 and GSE239916.

## 1 Introduction

The study of gene expression and its regulation is central to molecular biology, providing critical insights into cellular function and organismal development. A key challenge in this field is understanding how genetic information encoded in chromosomes is regulated and transcribed into mRNA, ultimately shaping gene expression (Bannister and Kouzarides 2011). Recent advancements in multimodal single-cell omics technologies have enabled the simultaneous profiling of chromatin accessibility and gene expression within individual cells. By integrating the paired single-cell ATAC sequencing (scATAC-seq) and single-cell RNA sequencing (scRNA-seq) data, these technologies provide promise to identify key regulatory elements including enhancers and promoters that influence gene expression in a cell-type-specific manner.

A series of studies have been proposed to investigate the relationship between scRNA-seq and scATAC-seq data using paired multi-omics information. For example, studies have focused on generating embeddings of cells based on both gene expression and chromatin accessibility data, demonstrating strong performance in cell-type discrimination (Cao *et al.* 2022, Tang *et al.* 2023, Zeng *et al.* 2023, Cao *et al.* 2024, Cheng *et al.* 2025, Cui *et al.* 2025). Previous studies have also explored the relationship between gene expression and chromatin accessibility through the lens of transcription factor (TF) motif analysis, aiming to construct more accurate gene regulatory networks by integrating both data types (Li *et al.* 2022, Bravo González-Blas *et al.* 2023, Loers and Vermeirssen 2024, Xu *et al.* 2025, Yuan and Duren 2025).

However, only a limited number of studies have focused on the identification of regulatory elements that influence gene expression (Granja *et al.* 2021, Singh *et al.* 2022, Hu *et al.* 2024, Liu *et al.* 2024, Mitra *et al.* 2024, Zhong *et al.* 2024, Saelens *et al.* 2025). Most of these methods rely on regression or predictive models to establish associations between chromatin accessibility peaks and expressed genes. While these models are useful, they often struggle to handle the inherently weak peak signals and may fail to fully detect certain important regulatory interactions between peaks and genes. Studying relationships among peaks and genes—such as peak–peak co-accessibility and gene–gene co-expression—may provide valuable information for capturing peak–gene associations more effectively. A key challenge we aim to address is how to integrate these interactions to enhance the accuracy of peak–gene relationship inference. Additionally, existing methods typically analyze peak–gene associations in an isolated manner, examining each gene independently. Such a fragmented approach fails to capture the global regulatory landscape between peaks and genes, limiting our understanding of the complex interplay within gene regulatory networks. Therefore, there is a need for a more integrative framework that captures the underlying structure of peak–gene interactions in a unified manner, enabling a comprehensive representation of regulatory influences.

In this study, we propose single-cell meta-Path based Omics EMbedding (scPOEM), a novel method that jointly embeds genes and chromatin accessibility peaks into a unified low-dimensional space. Different from existing network-based methods for integrating scATAC-seq and scRNA-seq data, such as SIMBA (Chen *et al.* 2024) and scMI (Cai *et al.* 2024), which connect peaks and genes indirectly via cells and primarily focus on cell-to-cell variation, scPOEM focuses on peak–gene associations within a group of homogeneous cells of the same cell type, enabling a more direct and biologically meaningful representation of *cis*-regulatory relationships. Specifically, scPOEM generates low-dimensional representations of genes and peaks to better characterize their regulatory interactions. The method first constructs a heterogeneous network incorporating peak–peak, peak–gene, and gene–gene relationships, and then employs an improved meta-path-based approach for heterogeneous network embedding. In the resulting low-dimensional space, a peak and a gene that are closer together are considered to exhibit a stronger regulatory association. Compared to other network embedding methods, scPOEM provides more stable and interpretable cross-modal integration. In benchmarking analysis, scPOEM demonstrates superior performance in detecting experimentally validated super-enhancers and in identifying Promoter Capture HiC (PCHiC) interactions, compared to existing regression-based methods. Furthermore, by clustering genes in the low-dimensional space and examining the closest peaks corresponding to each cluster of genes, scPOEM enables the exploration of relationships between gene and peak groups, offering new biological insights in peak–gene regulatory mechanisms. Meanwhile, with real data, by applying scPOEM to memory CD4+ T cells from the HIV-infected and control samples, we reveal significant differences in peak–gene relationships for disease-associated genes, providing new insights into how chromatin accessibility and gene regulation are altered.

## 2 Materials and methods

### 2.1 Quality control and preprocessing

scPOEM uses paired scRNA-seq and scATAC-seq data as input. We adhered to the standardized QC procedures for both scRNA-seq and scATAC-seq datasets. Starting with the quality control (QC) for cells, cells with excessively low or high RNA and ATAC counts, as well as those with mitochondrial read proportions exceeding 20%, were excluded. For the feature-level filtering, we retained peaks located on “standard” chromosomes and genes with valid annotations. We also removed peaks and genes from the X, Y, and mitochondrial chromosomes, as well as those located within blacklisted genomic regions. Peaks detected in fewer than five cells and genes detected in fewer than 1% cells are also removed. The scRNA-seq data were sequentially library-size normalized, log transformed and scaled. Then 3000 highly variable genes (HVGs) were selected for downstream analysis. The scATAC-seq peak counts were subsequently transformed using TF-IDF. The *Seurat* (v5.1) (Hao *et al.* 2024) and *Signac* (v1.13) (Stuart *et al.* 2021) R packages are used to process the scRNA-seq and scATAC-seq data in practice.

### 2.2 The scPOEM workflow

**Figure 1.**
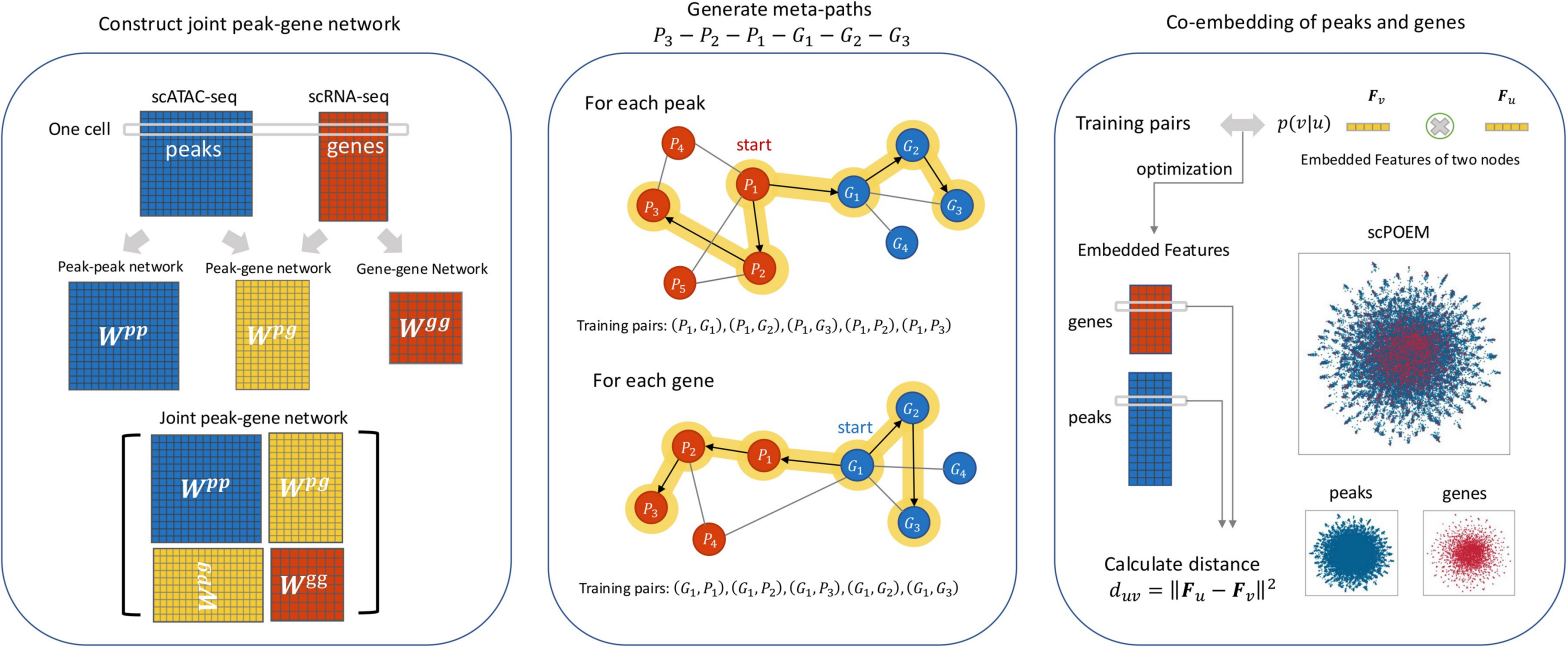
The scPOEM workflow. It includes constructing the joint peak–gene network, generating meta-paths through heterogeneous random walks, and co-embedding peaks and genes.

There are three main steps of scPOEM as shown in Fig. 1. After preprocessing, scPOEM first constructs peak–peak, peak–gene, and gene–gene networks, forming a joint peak–gene network. Then based on this joint network, scPOEM generates meta-paths of the scheme $P_{3}-P_{2}-P_{1}-G_{1}-G_{2}-G_{3}$ via random walk, using different transition matrices for each type of link. Finally, based on the training dataset derived from the meta-paths, the heterogeneous network embedding method creates embedded features for each gene and peak in the same low-dimensional space. For each gene, scPOEM calculates its distance to each peak, with the nearest peaks considered to have a potential functional relationship with the gene and influence its expression. The embedding step of scPOEM is inspired by the metapath2vec algorithm (Dong *et al.* 2017), but differs significantly by generating meta-paths through a regulatory-based process that explores higher order relationships between peaks and genes. The details of the three steps are described in the following subsections.

### 2.3 Constructing the joint network of peaks and genes

For n cells, suppose $X\in R^{n\times p}$ is the matrix for the scATAC-seq data with p peaks, and $Y\in R^{n\times q}$ is the count matrix for the scRNA-seq data with q genes. The scPOEM first builds the joint regulatory network of peaks and genes based on X and Y, which consists of three parts—peak–peak network, peak–gene network, and gene–gene network. This joint network belongs to the heterogeneous network as its nodes include two types of entities.

#### 2.3.1 Peak–peak network construction

Since Cicero (Cusanovich *et al.* 2018, Pliner *et al.* 2018) effectively utilizes single-cell chromatin accessibility data to predict regions of the genome that are more likely to be in physical proximity in the nucleus, enabling the identification of potential enhancer-promoter pairs and the *cis*-regulatory architecture, we utilized this method to construct peak–peak network. The basic workflow of Cicero involves converting the count matrix X into a binary matrix, performing dimensionality reduction on cells using UMAP, aggregating cells with KNN, and finally applying a graphical Lasso model to infer co-expression patterns between pairs of peaks. To focus solely on the degree of association between peaks, irrespective of directionality, we took the absolute value of all pairwise relationships and recorded the results in the matrix $W^{pp}\in R^{p\times p}$, which is symmetric with all diagonal entries equal to 0. The ($i_{1}$, $i_{2}$)th entry of $W^{pp}$ indicates the co-expression level between the $i_{1}$th peak and the $i_{2}$th peak.

#### 2.3.2 Gene–gene network construction

Many methods have been proposed to construct the gene–gene regulatory network (Pratapa *et al.* 2020), and we select to use the principal component regression method, since its good performance and high efficiency as shown in existing works (Yang *et al.* 2023a,b). For the jth gene, use $Y_{j}$ to denote the jth column of Y and use $Y_{-j}\in R^{n\times (q-1)}$ to denote the matrix of Y without the jth column. Principal component analysis is applied to $Y_{-j}$, and the first five leading principal components are maintained to construct the new feature matrix $U\in R^{n\times 5}$. Denote the corresponding loading matrix for these five principal components by $V^{j}\in R^{(q-1)\times 5}$ that ${{(V}^{j})}^{T}V^{j}=I_{5}\;\mathrm{and}\;U^{j}=Y_{-j}V^{j}$. Then we use the new feature matrix to regress $Y_{-j}$ and the estimated coefficient by the ordinary least square method is ${\hat{\alpha }}^{j}={\left(U^{T}U\right)}^{-1}U^{T}Y_{j}\in R^{5}$. Compute ${\hat{\beta }}^{j}=V^{j}{\hat{\alpha }}^{j}\in R^{q-1}$, which reflects the effects of other genes to the jth gene. After ${\hat{\beta }}^{j},\;j=1,\ldots ,q$ are obtained, we construct $W^{gg}\in R^{q\times q},\;$whose jth column is consisted by ${\hat{\beta }}^{j}$ and a zero value on the jth entry. $W^{gg}$ is also taken the absolute value and symmetrized, and only values greater than the 95th percentile were retained to highlight the strongest associations. $W^{gg}$ can be used to reflect the strength of gene–gene interactions that a large value on its ($j_{1}$, $j_{2}$)th entry indicates that the $j_{1}$th gene and $j_{2}$th gene are highly correlated and may regulate each other.

#### 2.3.3 Peak–gene network construction

As discussed in Section 1, learning the relationship between peaks and genes is still in an early stage, and which method provides the best performance is still an uncontained question. Therefore, instead of using only one method, we consider using three different methods, including Lasso regression (Tibshirani 1996), random forest (RF) (Breiman 2001), and XGBoost (Chen and Guestrin 2016) to analyze the interactions between peaks and genes. For each method, we construct a matrix $W^{pg}\in R^{p\times q}$ to reflect the interaction of peaks and genes and obtain three different matrices denoted by $W_{\mathrm{Lasso}}^{pg}$, $W_{\mathrm{RF}}^{pg}$, and $W_{\mathrm{XGBoost}}^{pg}$. Later on, when constructing the embedding, we will use the ensemble strategy to integrate the information of three matrices together during the embedding progress. Each method constructs its $W^{pg}$ via a genewise progress. For the jth gene, we use peaks within the 100 kbp upstream and downstream of the gene as explanatory variables to build prediction models for the gene expression $Y_{j}$. The detailed instructions of using three methods are included in the Supplementary Material, available as supplementary data at *Bioinformatics* online.

### 2.4 Generating meta-paths

#### 2.4.1 Meta-path scheme

The main goal of our embedding is to extracting the peak–gene relationship in the joint regulatory network. Besides the direct links between peaks and genes in the network, we emphasize that the higher order links between peaks and genes in the network may also include information. Focusing on an existing peak–gene link between the peak $P_{1}$ and the gene $G_{1}$, denoted by $P_{1}-G_{1}$, there are two potential second-order links between peak and gene:

1. A peak ($P_{2}$) is a potential regulatory of $G_{1}\;$if it co-expresses with $P_{1}$. This path is $P_{2}-P_{1}-G_{1}$.
2.
$P_{1}$ is a potential regulatory of a gene ($G_{2}$) if $G_{1}$ directly regulates $G_{2}$. This path is $P_{1}-G_{1}-G_{2}$.

Similarly, for the third-order links, we consider the path $P_{3}-P_{2}-P_{1}-G_{1}$ to include the potential relationship between $P_{3}$ and $G_{1}$, and the path $P_{1}-G_{1}-G_{2}-G_{3}$ to include the potential relationship between $P_{1}$ and $G_{3}$. The above relationships can be combined into one meta-path scheme $P_{3}-P_{2}-P_{1}-G_{1}-G_{2}-G_{3}$. Within each meta-path, the peak $P_{1}$ is shown to related to $P_{2}$, $P_{3}$, $G_{1}$, $G_{2}$, and $G_{3}$, which results in five node pairs in the heterogenous network and will be used as training datasets for embedding learning. Similarly, the gene $G_{1}$ is shown to be related to $P_{1}$, $P_{2}$, $P_{3}$, $G_{2}$, and $G_{3}$, resulting in five training node pairs. Therefore, we will focus on generating samples of this meta-path scheme for each gene and peak and construct training node pairs. While higher order links beyond the third order can be incorporated in the similar manner, they provide marginal improvements by our experiments. A detailed discussion on the impact of meta-path length is provided in Supplementary Material, available as supplementary data at *Bioinformatics* online.

#### 2.4.2 Construct transitions matrices

To sample meta-paths, we consider the way to sample the next node from a given node in the joint network. As there are two kinds of nodes in the network, $2^{2}=4$ distinct node sampling strategies should be specified. With $W^{pp}$, $W^{gg}$, $W_{\mathrm{Lasso}}^{pg}$, $W_{\mathrm{RF}}^{pg}$, and $W_{\mathrm{XGBoost}}^{pg}$, we construct four transitions matrices to guide the four sampling scenarios, respectively, as follows. To begin with, for each W, we clip its non-zero values at the 10th and 90th percentiles to avoid extreme values.

1. Sample a peak from a peak: $W^{pp}$ is normalized by row to assure that each row of $W^{pp}$ equal to 1, denoted by $P^{pp}$. Then $P^{pp}$ is the transition matrix of peaks.
2. Sample a gene from a gene: Similarly, $W^{gg}$ is normalized by row and denoted by $P^{gg}$. $P^{gg}$ is the transition matrix of genes.
3. Sample a gene from a peak: $W_{\mathrm{Lasso}}^{pg}$, $W_{\mathrm{RF}}^{pg}$, and $W_{\mathrm{XGBoost}}^{pg}$ are normalized by row and denoted by $P_{\mathrm{Lasso}}^{pg}$, $P_{\mathrm{RF}}^{pg}$, and $P_{\mathrm{XGBoost}}^{pg}$, respectively. Then we construct $P^{pg}=1/3(P_{\mathrm{Lasso}}^{pg}+P_{\mathrm{RF}}^{pg}+\;P_{\mathrm{XGBoost}}^{pg})$, which is the transition matrix from peaks to genes.
4. Sample a peak from a gene: ${\left(W_{\mathrm{Lasso}}^{pg}\right)}^{T}$, ${\left(W_{\mathrm{RF}}^{pg}\right)}^{T}$, and ${\left(W_{\mathrm{XGboost}}^{pg}\right)}^{T}$ are normalized by row and denoted by $P_{\mathrm{Lasso}}^{gp}$, $P_{\mathrm{RF}}^{gp}$, and $P_{\mathrm{XGBoost}}^{gp}$, respectively. Then we construct $P^{gp}=1/3(P_{\mathrm{Lasso}}^{gp}+P_{\mathrm{RF}}^{gp}+P_{\mathrm{XGBoost}}^{gp})$, which is the transition matrix from genes to peaks.

$P^{pg}$ and $P^{gp}$ are constructed via the ensemble strategy. We define the SampleNode function as

**function**  SampleNode(node i, transition matrix P)

   prob_dist←P[i, :]

   j ← sample_from_distribution(prob_dist)

  **return**  j

#### 2.4.3 Training node pair construction

Algorithm 1 includes the specific way to generate meta-paths from peak and gene aspects respectively via random walks. We emphasize that each node pair is also associated with a weight of $e^{-0.1k}$, where k∈{0,1,2} denotes the number of nodes between two nodes in the meta-path. The output set of training node pairs is denoted by E.

Algorithm 1
**Input:** Transitions matrices $P^{pp}$,$P^{gg}$, $P^{pg}$ and $P^{gp}$; The number of loops T; Three empty lists $L_{\mathrm{peak}}$, $L_{\mathrm{gene}}$, and E.

**For**  loop=1, 2,…, T do

 **Foreach** peak $P_{1}$ in 1:p do

  $G_{1}\leftarrow \mathrm{SampleNode}(P_{1},\;P^{pg})$; $G_{2}\leftarrow \mathrm{SampleNode}(G_{1},\;P^{gg})$;

  $G_{3}\leftarrow \mathrm{SampleNode}(G_{2},\;P^{gg})$; $P_{2}\leftarrow \mathrm{SampleNode}(P_{1},\;P^{pp})$;

$\;\;P_{3}\leftarrow \mathrm{SampleNode}(P_{2},\;P^{pp})$ ;

  $L_{\mathrm{peak}}\leftarrow L_{\mathrm{peak}}+\{P_{3}-P_{2}-P_{1}-G_{1}-G_{2}-G_{3}\}$;

  $E\leftarrow E+(P_{1},\;G_{1},\;1)$; $E\leftarrow E+(P_{1},\;P_{2},\;1)$;

  $E\leftarrow E+(P_{1},\;G_{2},\;e^{-0.1})$; $E\leftarrow E+(P_{1},\;P_{3},\;e^{-0.1})$;

  $E\leftarrow E+(P_{1},\;G_{3},\;e^{-0.2})$;

 **EndFor**

 **Foreach** gene $G_{1}$ in 1:q do

  $G_{2}\leftarrow \mathrm{SampleNode}(G_{1},\;P^{gg});\;G_{3}\leftarrow \mathrm{SampleNode}(G_{2},\;P^{gg})$;

  $P_{1}\leftarrow \mathrm{SampleNode}(G_{1},\;P^{gp})$; $P_{2}\leftarrow \mathrm{SampleNode}(P_{1},\;P^{pp});$

  ${\;P}_{3}\leftarrow \mathrm{SampleNode}(P_{2},\;P^{pp})$;

  $L_{\mathrm{gene}}\leftarrow L_{\mathrm{gene}}+\{P_{3}-P_{2}-P_{1}-G_{1}-G_{2}-G_{3}\}$.

  $E\leftarrow E+(G_{1},\;P_{1},\;1)$; $E\leftarrow E+(G_{1},\;G_{2},\;1)$;

  $E\leftarrow E+(G_{1},\;P_{2},\;e^{-0.1})$; $E\leftarrow E+(G_{1},\;G_{3},\;e^{-0.1})$;

$\;\;E\leftarrow E+(G_{1},\;P_{3},\;e^{-0.2})$

 **EndFor**

**EndFor**

**Output:** The meta-path lists $L_{\mathrm{peak}}$ and $L_{\mathrm{gene}}$, and the training node pair set E.

### 2.5 Learning co-embedding of peaks and genes

Given the training node pair set E, we construct *d*-dimensional embeddings of peaks and genes, represented as $F\in \;R^{(p+q)\times d}$. For the rth tuple $\left(u_{r},\;v_{r},\;w_{r}\right)$ in E, the embeddings of $u_{r}$ and $v_{r}$ are given by the corresponding row vectors of F, denoted as $F_{u_{r}}\in \;R^{d}$ and $F_{v_{r}}\in \;R^{d}$, respectively. To train the embedding matrix, we maximize the product of the probability $p(v_{r}|u_{r})$ for each pair, which have a form of


$$p(v_{r}|u_{r})=\frac{e^{F_{v_{r}}\cdot F_{u_{r}}}}{\sum_{c\in V}e^{F_{c}\cdot F_{u_{r}}}},$$

where · denotes the inner product of two vectors and V is the vertex set that includes all genes and peaks. Since the dominant of $p(v_{r}|u_{r})$ is hard to calculate, we apply the negative sampling technique (Mikolov *et al.* 2013) that for each pair ($u_{r},\;v_{r}$), we randomly sample M negative nodes, denoted by $c_{r}^{1},\ldots ,c_{r}^{M}$ from the set of all peaks and genes. The final objective function to maximize can be written as


$$O(F)=\sum_{r=1}^{\left|E\right|}w_{r}(\log\sigma (F_{v_{r}}\cdot F_{u_{r}})+\sum_{m=1}^{M}\log\sigma (-F_{c_{r}^{m}}\cdot F_{u_{r}})),$$

where $\sigma (x)=\frac{1}{1+e^{-x}}$ is the sigmoid function and |E| is the number of node pairs in E. This optimization problem can be solved by block stochastic descent easily in the similar way of the meta-path2vec algorithm.

Using the low-dimensional representation F, we calculated the Euclidean distance $d_{uv}$ between each pair, including peaks and peaks, peaks and genes, as well as genes and genes in the unified space as $d_{uv}={\left\|F_{u}-F_{v}\right\|}^{2}$. For each gene, nearby peaks at a short distance suggest potential functional relationships.

### 2.6 Datasets

Four groups of paired scATAC-seq and scRNA-seq datasets from three studies are used in our study. These studies were performed by different laboratories, thereby ensuring independent experimental conditions. All paired datasets are derived from the “10× Genomics Chromium Single Cell Multiome ATAC + Gene Expression” multiomics technology. Table 1 gives a summary of these datasets. The PBMC dataset was downloaded from 10× Genomics. Cell types were annotated using information from TRIPOD (Jiang *et al.* 2022) with Naive CD4+ T cells selected for analysis. The BMMC dataset is obtained from GEO (GSE194122) (Luecken *et al.* 2021), and we focus on the donors of site 1 and the cell type of CD8+ T as it has the largest number of cells. The HIV datasets, obtained from GEO (GSE239916) contains memory CD4+ T cell samples from infected and uninfected donors (Wei *et al.* 2023). We selected two representative samples: the healthy case (denoted by HC) and the early viremia-state HIV-infected case YW8 (denoted by HIV) to compare regulatory mechanisms between immunologically naive and initial viral exposure states. By applying the proposed method to the data of each condition, we can investigate the ability of our method to uncover disease-associated regulatory mechanisms.

**Table 1.** Summary of real-data applications of analysis.^a^

Study	Dataset	Source	Cell type	Number of peaks after QC	Number of cells after QC
1	PBMC	10× Genomics	Naive CD4+ T	67 337	1194
2	BMMC	GSE194122	CD8+ T	80 903	3180
3	HC	GSE239916	Memory CD4+ T	62 585	10 262
	HIV	GSE239916	Memory CD4+ T	62 158	6417

a The dataset PBMC was obtained from https://www.10xgenomics.com/datasets/pbmc-from-a-healthy-donor-granulocytes-removed-through-cell-sorting-10-k-1-standard-1-0-0.

### 2.7 Performance evaluation

For evaluation, we constructed a d = 100-dimensional embedding for each gene and peak using scPOEM for each dataset. The computing time of scPOEM for different datasets is included in the Supplementary Material, available as supplementary data at *Bioinformatics* online. We evaluated the overall effectiveness of scPOEM from four aspects. First, to assess its ability to identify super-enhancer-associated peaks, we utilized the SEA database (Chen *et al.* 2020) to compare scPOEM with Lasso, RF, and XGBoost in detecting peak–gene pairs within 200 kb of the gene body by precision and recall. We also examined whether peaks overlapping with super-enhancers tend to be assigned closer distances to their target genes in the latent space using boxplots. To evaluate scPOEM’s effectiveness in identifying PCHiC interactions, we analyzed a publicly available PCHiC dataset (Javierre *et al.* 2016) to assess the overlapping number between the closest peak–gene pairs inferred by scPOEM and PCHiC interaction regions. Additionally, to evaluate scPOEM’s effectiveness in identifying gene groups with regulatory mechanisms, we clustered genes based on the embedded features and identified a group of closest peaks for each gene subgroup. We then applied gene enrichment analysis via EnrichR (Kuleshov *et al.* 2016) and peak set enrichment via GREAT (McLean *et al.* 2010) to explore the potential regulatory roles within each cluster. Finally, to assess scPOEM’s ability to identify disease-associated regulatory factors, we constructed a new gene–gene network using embedded features for each of the healthy and HIV-infected datasets and employed the manifold alignment technique proposed by scTenifoldNet (Osorio *et al.* 2020, Zhong et al. 2025) to detect differentially regulated genes between the two conditions. We further analyzed the regulations of the top differentially regulated genes and their closest peaks. A more detailed description of the performance evaluation is included in the Supplementary Material, available as supplementary data at *Bioinformatics* online. All external resources used for evaluation are independent of the four scRNA-seq and scATAC-seq datasets, ensuring the validity and robustness of our entire evaluation process.

## 3 Results

We applied scPOEM to four datasets and generated 100 embedding features for each peak and gene. The same parameter settings were used across all experiments: during co-embedding learning, we employed stochastic gradient descent with a batch size of 32, a learning rate of 0.1, and early stopping after 100 training epochs. In each epoch, we sampled five random meta-paths per node and drew five negative samples for each positive peak–gene pair within a meta-path. The detailed numerical values of the embedding features are provided in the Supplementary Materials, available as supplementary data at *Bioinformatics* online.

### 3.1 scPOEM provides robust co-embedding and better mixing of peaks and genes

**Figure 2.**
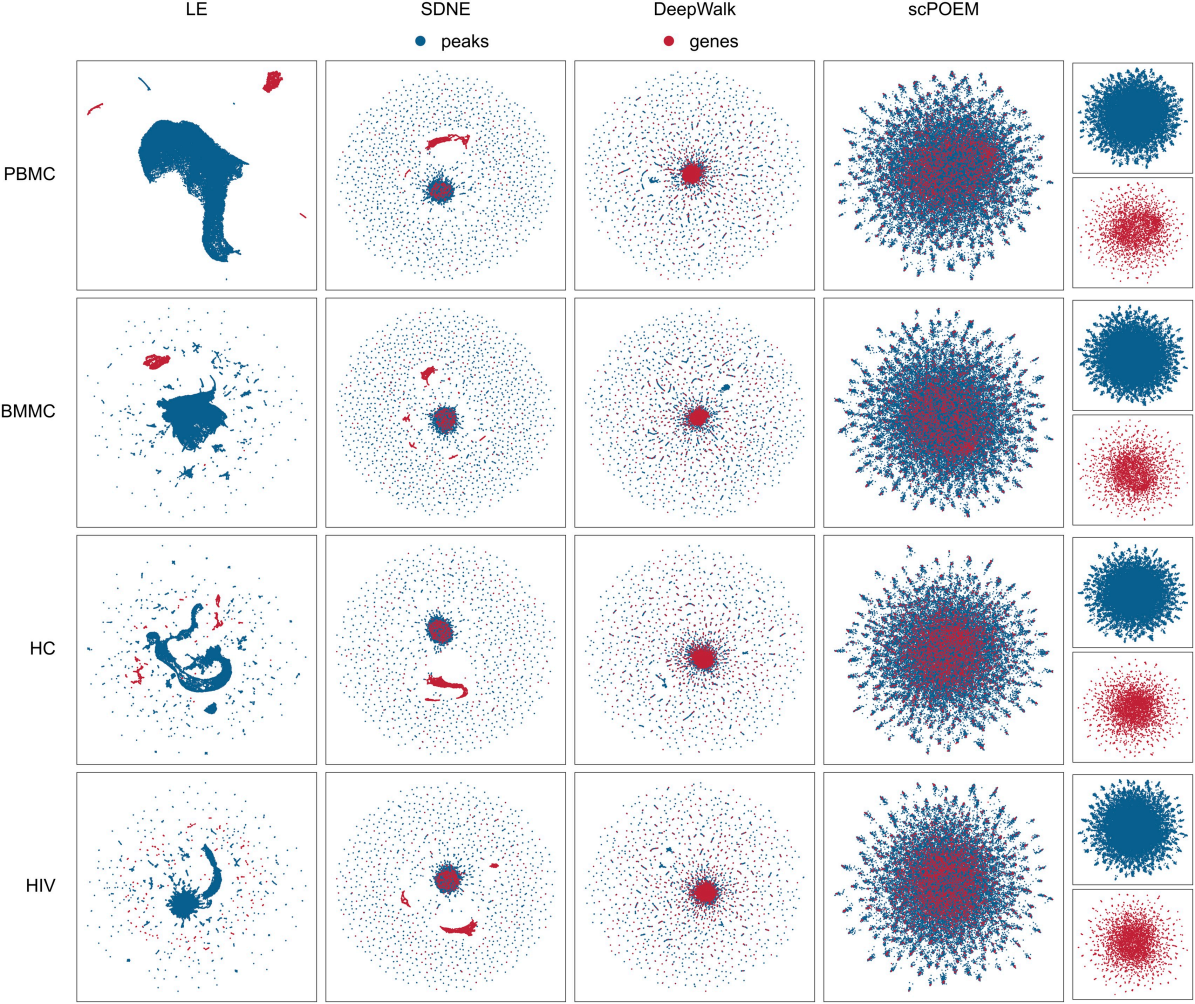
Two-dimensional UMAP visualization of 100 embedded features generated by Laplacian Eigenmaps, SDNE, DeepWalk, and scPOEM for four datasets.

Figure 2 displays the two-dimensional UMAP projection (Becht *et al.* 2018) of the 100 embedded features generated by scPOEM. For comparison, three popular network embedding methods—Laplacian Eigenmaps (Belkin and Niyogi 2003), Structural Deep Network Embedding (SDNE) (Wang *et al.* 2016), and DeepWalk (Perozzi *et al.* 2014)—were also applied to the joint network of peaks and genes, with their results visualized using UMAP. For each dataset, it is clear that scPOEM achieves the best cross-modality integration of scATAC-seq and scRNA-seq data, resulting in a robust and improved mixing of genes and peaks that reflects their shared regulatory space. In all cases, scPOEM produced a more dispersed distribution, positioning genes between peaks to better reflect the biological organization of regulatory elements. This structure enhances the interpretability of peak–gene relationships and aids in identifying potential regulatory interactions. In contrast, Laplacian Eigenmaps tended to separate peaks and genes into distinct clusters, failing to integrate information from both modalities effectively. While SDNE and DeepWalk outperformed Laplacian Eigenmaps in cross-modal integration, their embeddings are overly compact. Most genes and peaks are densely clustered in the center, making it difficult to infer regulatory interactions.

Sensitivity analysis is conducted to assess the stability of our method in providing low-dimensional embeddings. For example, using the PBMC data, we created two random subsamples of 90% of the cells and applied the scPOEM method to each subsample. For each gene, we compared the ranks of peaks based on their distance to the gene in both subsamples via Spearman correlations. The Spearman correlations for all genes range from 0.77 to 0.81, with a median value of 0.802. These results indicate that the ranks of peaks relative to genes are stable across different data subsamples, further demonstrating that scPOEM provides robust co-embedding of peaks and genes.

### 3.2 scPOEM demonstrates superior performance in identifying super-enhancer regions and PCHiC interactions compared to regression methods

**Figure 3.**
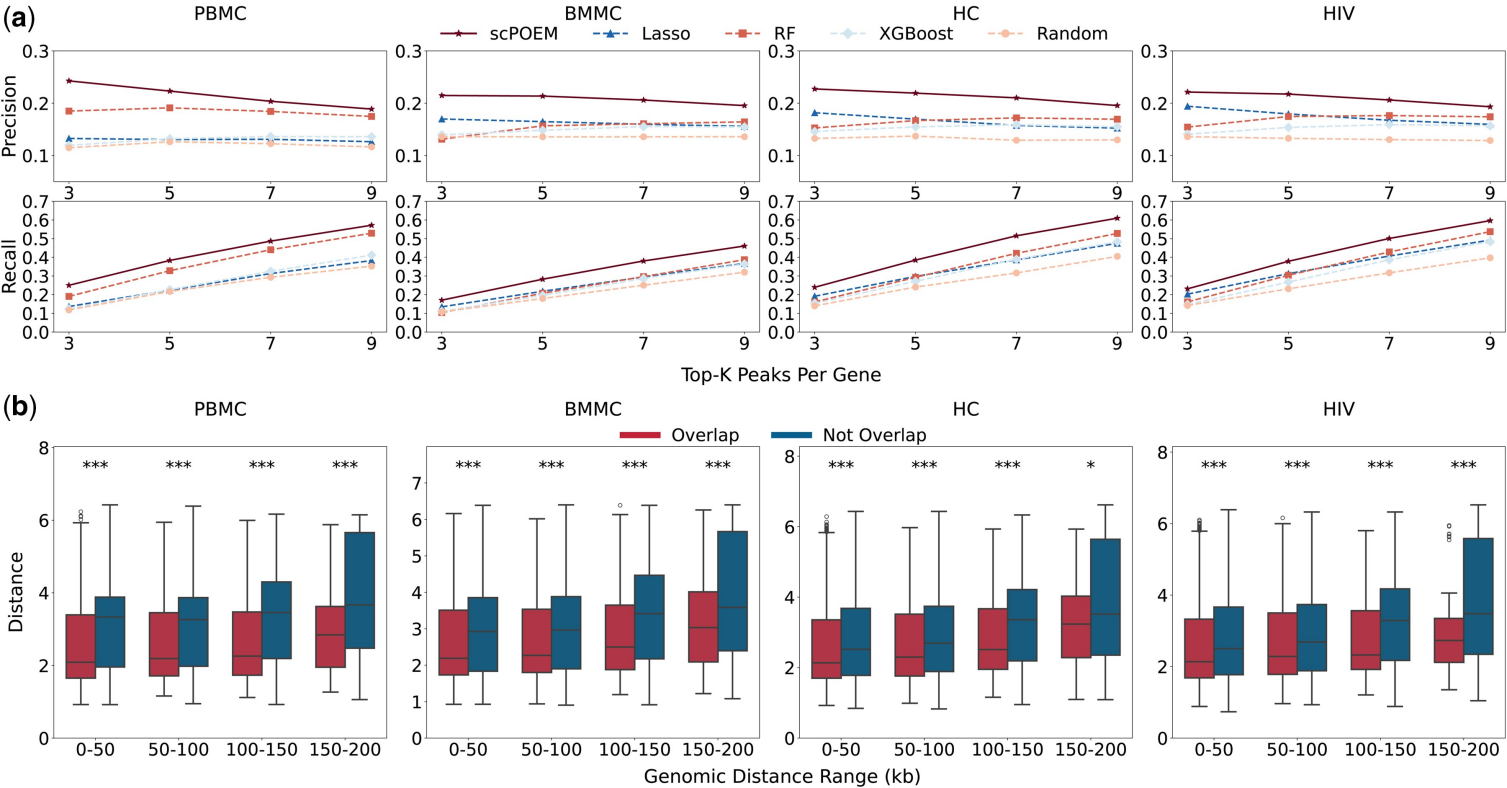
Performance in identifying super-enhancer regions. (a) Average precision and recall of the compared methods for detecting super-enhancer-associated peaks for each gene across four datasets. (b) Boxplots of embedded distances for peak–gene pairs within different neighborhood windows, comparing peaks overlapped and not overlapped with super-enhancer regions.

We investigated whether nearby peaks and genes in the embedded space exhibit more realistic regulatory information and reflect chromatin–gene relationships. We first assessed the performance of scPOEM in identifying super-enhancer regions for each gene in Fig. 3. Figure 3a shows the number of super-enhancers covered by the top peak–gene pairs detected by scPOEM, Lasso, RF, XGBoost, and random selection. scPOEM achieves overall higher precision and recall compared to Lasso, RF, and XGBoost, indicating that scPOEM effectively captures key regulatory interactions within the joint regulatory network of genes and peaks. Figure 3b presents boxplots of the distances between genes and peaks overlapping with super-enhancer regions, which are generally lower than those of peaks outside super-enhancer regions. This result suggests that scPOEM assigns closer embeddings to functionally related regulatory elements, effectively capturing underlying regulatory interactions.

**Figure 4.**
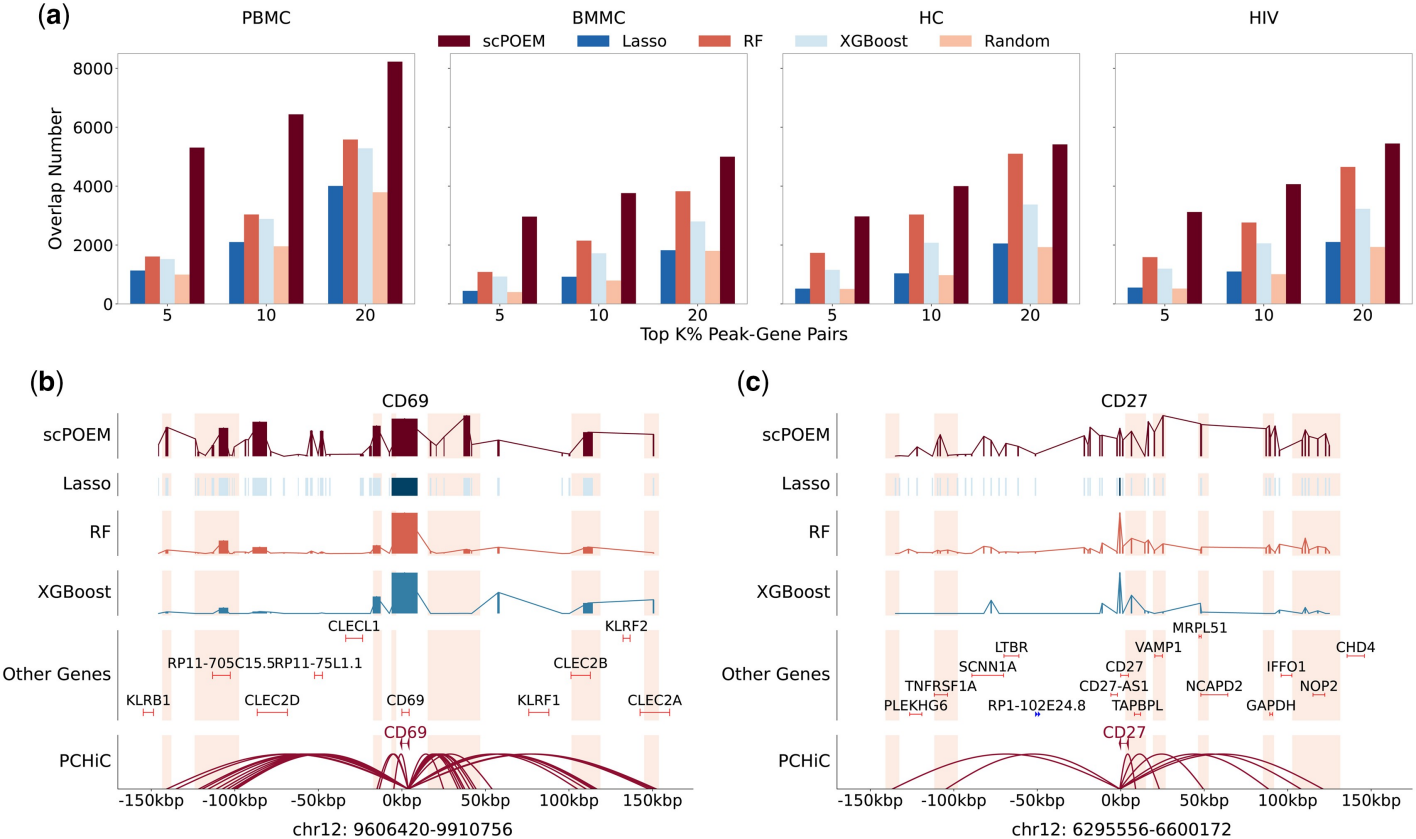
Performance in identifying PCHiC interactions. (a) The number of overlaps between PCHiC interactions and different percentages of top peak–gene pairs identified by scPOEM and other methods. (b–c) Examples of scPOEM interpretation for the CD69 gene in the PBMC dataset and the CD27 gene in the BMMC dataset. The curves show the importance scores of different methods across peaks in the upstream and downstream of the target gene, with highlighted regions indicating PCHiC interactions. For scPOEM, the importance score is calculated as exp(-dist), where dist is the distance between the peak and the target gene in the low-dimensional space.

Next, we evaluated the performance of scPOEM in identifying PCHiC interactions, as shown in Fig. 4. Figure 4a displays the number of PCHiC interactions matched by the top peak–gene pairs for each method. scPOEM consistently achieves higher overlapping than other methods across different settings, demonstrating its ability to capture key regulatory interactions within the joint regulatory network of genes and peaks. Two examples are provided in Fig. 4b to illustrate the superiority of scPOEM. For the PBMC dataset, CD69 is a key regulator of T cell function, particularly in naive CD4+ T cells, where it influences differentiation and helps maintain immune homeostasis (de la Fuente *et al.* 2014). scPOEM assigns higher importance scores to peaks in PCHiC interaction regions than other methods for CD69. For the BMMC dataset, CD27 is a critical marker gene for CD8+ T cells, promoting their survival, facilitating memory formation, and distinguishing early stage from terminally differentiated subsets, as terminally differentiated effector CD8+ T cells often lose CD27 expression (Hendriks *et al.* 2000). Similarly, scPOEM outperforms other methods in identifying PCHiC interactions for CD27.

By performing ablation experiments, we found that the improvement from using the ensemble strategy is modest compared to using only RF or XGBoost in scPOEM. Therefore, users may choose to use a single method to reduce computational time. More details are provided in the Supplementary Material, available as supplementary data at *Bioinformatics* online.

### 3.3 scPOEM provides consistent and enhanced group-level analysis for genes and related peaks

**Figure 5.**
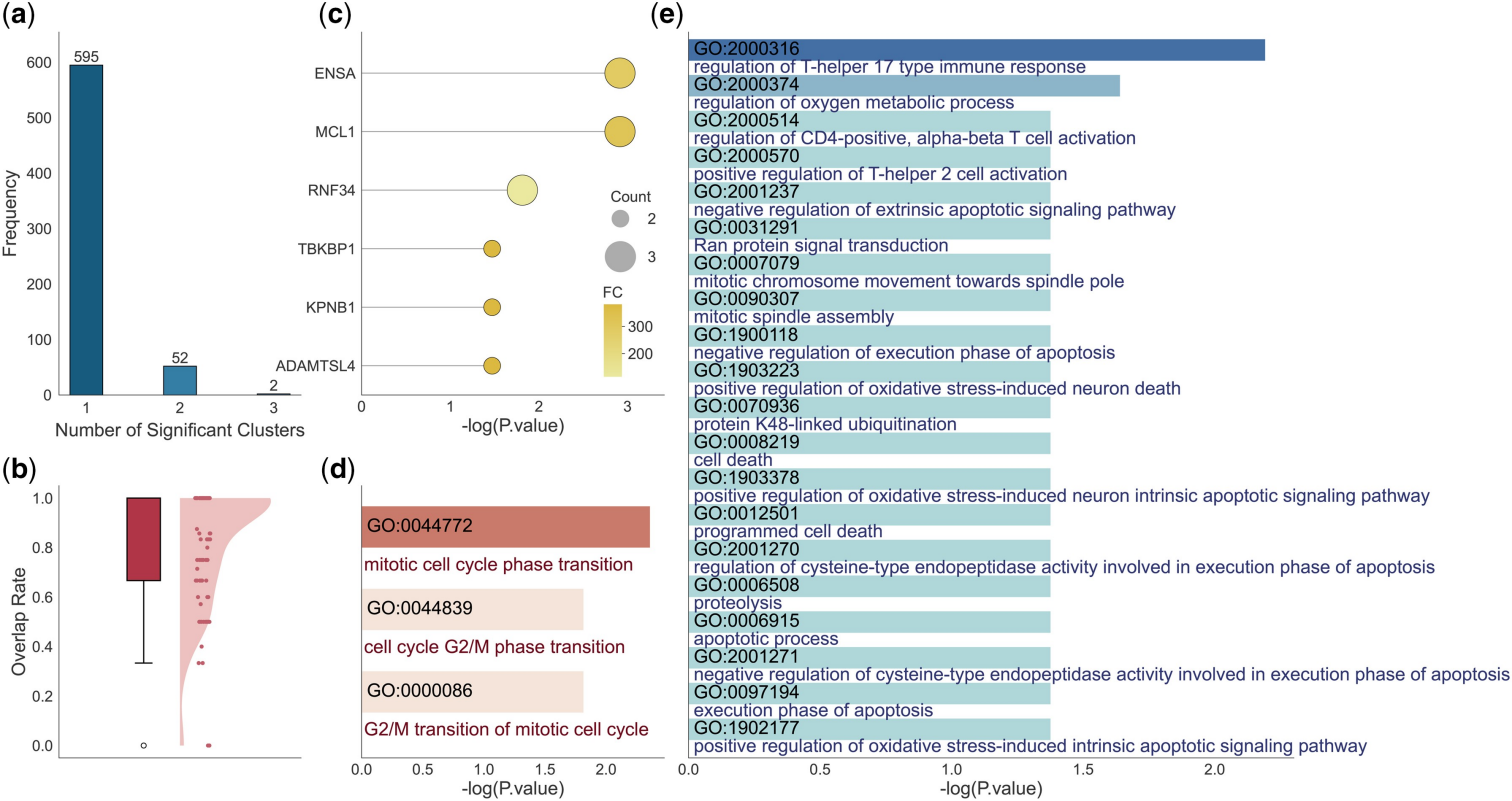
Enrichment results for gene and assigned peak groups in the PBMC dataset. (a) Distribution of subgroup counts related to one enriched pathway. (b) Percentage of genes enriched by peaks that are covered by the gene subgroup (considering only those within the retained HVGs). (c–e) Results for an example gene group (the #49 gene group) and its assigned peaks: (c) List of enriched genes by peaks. (d, e) Lists of enriched pathways by genes and peaks via EnrichR and GREAT, respectively.

Using the PBMC dataset as an example, it contains 3000 HVGs and 67 337 peaks after preprocessing. We grouped genes into 100 subgroups of sizes 10–50 and assigned each subgroup a set of peaks based on the embedded features. The average number of related peaks per subgroup is 45.18. Within each subgroup, we conducted gene enrichment analysis, identifying a total of 705 distinct pathways. Figure 5a shows the number of gene subgroups associated with each pathway, revealing that most pathways are enriched in only one subgroup, indicating that scPOEM effectively groups functionally related genes together. We then performed peak enrichment for each group of peaks, first identifying enriched genes, which were then mapped to enriched pathways. As shown in Fig. 5b, the enriched genes identified by peaks largely overlap with subgroup genes. Figure 5c presents an example of subgroup #49 where ENSA, MCL1, RNF34, TBKBP1, KPNB1, and ADAMTSL4 are enriched by peaks. Notably, ENSA and RNF34 are among the top 3000 HVGs, but only ENSA is assigned to the gene group. Although RNF34 is not included in this group, our approach still successfully associates it with the relevant regulatory context. RNF34 is located in a closely related subgroup—the third nearest—highlighting our method’s sensitivity in capturing functionally connected genes beyond the HVG set.

Subgroup genes and those identified by peaks in the subgroup are functionally related and often provide complementary insights into gene functions. For example, Figure 5d and e shows the pathways identified by genes and peaks in subgroup #49. Gene enrichment highlighted key cell cycle-related pathways such as “mitotic cell cycle phase transition” (GO:0044772) and “G2/M transition of mitotic cell cycle” (GO:0000086). Similarly, peak-based enrichment revealed a broader set of functionally related pathways, including “mitotic spindle assembly” and “mitotic chromosome movement towards spindle pole,” underscoring active cell proliferation. Additionally, immune-related pathways such as “regulation of T-helper 17 type immune response” and “regulation of CD4-positive, alpha-beta T cell activation” were identified, reflecting the specific identity and functional state of naive CD4+ T cells (Dowling *et al.* 2018). Furthermore, apoptotic and stress response pathways found in the peak-based results, such as “execution phase of apoptosis” and “positive regulation of oxidative stress-induced intrinsic apoptotic signaling pathway,” align with the tightly regulated survival mechanisms in immune cells. Some enriched apoptotic pathways, including “negative regulation of cysteine-type endopeptidase activity involved in execution phase of apoptosis,” “apoptotic process,” “regulation of cysteine-type endopeptidase activity involved in execution phase of apoptosis,” “negative regulation of execution phase of apoptosis,” “negative regulation of extrinsic apoptotic signaling pathway,” and “execution phase of apoptosis” work in tandem with mitosis to maintain cellular turnover, where uncontrolled mitosis leads to proliferation, while apoptosis ensures proper cell clearance (Topham and Taylor 2013). These findings validate the reliability of co-embedding peaks and genes and highlight the advantage of leveraging both to identify significant regulatory pathways over using genes alone. The gene and peak lists and their enrichment results for the remaining subgroups are included in the Supplementary Material, available as supplementary data at *Bioinformatics* online.

### 3.4 scPOEM reveals significant differences in peak–gene regulatory interactions between HC and HIV conditions

**Figure 6.**
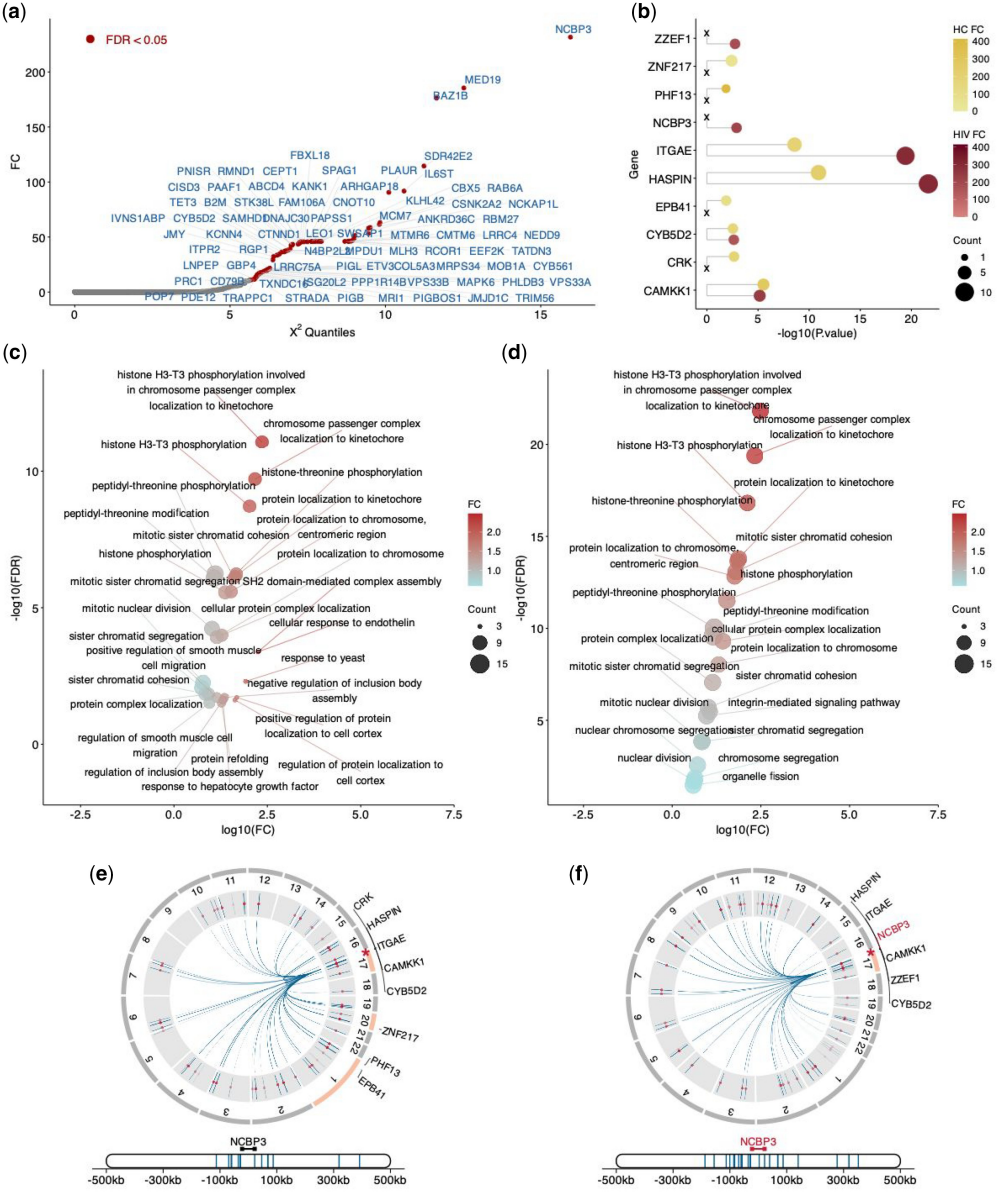
Differential analysis of regulatory gene networks between HC and HIV conditions. (a) Differentially regulated genes identified by scPOEM using the manifold alignment technique. (b) Enriched genes by the 100 nearest peaks of the NCBP3 gene for both conditions using GREAT. (c, d) Enriched pathways by the 100 nearest peaks of the NCBP3 genes for both conditions. (e, f) Visualization of the NCBP3 gene, its related peaks, and the enriched genes by peaks on chromatins in both conditions.

We applied the differential analysis described in performance evaluation to identify differentially regulated genes between HC and HIV conditions via scPOEM, as shown in Fig. 6a. NCBP3 ranked first among these genes. NCBP3 is an adapter protein that links capped RNAs (m7GpppG-capped RNA) to NCBP1/CBP80, playing a critical role in inhibiting virus growth (Gebhardt *et al.* 2015). Focusing on NCBP3, we selected its nearest 100 peaks in the embedded space for both conditions and conducted enrichment analysis using GREAT. The enriched genes for both conditions are shown in Fig. 6b. We found that NCBP3 is among the enriched genes in the HIV condition but not in HC, suggesting enhanced regulatory activity targeting this gene during HIV infection compared to healthy states. In terms of enriched pathways shown in Fig. 6c and d, the “integrin-mediated signaling” pathway was specifically identified in the HIV dataset but absent from the HC dataset, highlighting its critical role in HIV pathogenesis. This finding aligns with the known dependence of HIV on cell adhesion molecules for viral entry and dissemination. Specifically, integrins such as α4β7 and LFA-1 act as co-receptors for HIV-1 entry, promote virological synapse formation, and regulate immune cell trafficking that are activated during HIV infection (Arthos *et al.* 2008). These results validate scPOEM’s ability to project genes and related peaks to close locations in the co-embedding space.

Figure 6e and f visualize NCBP3, its 100 nearest peaks, and the enriched genes associated with these peaks in each condition. These peaks may be located outside the neighborhood regions of NCBP3 or even on different chromosomes. In the HIV dataset, the nearest peaks are primarily found within the same chromatin as NCBP3, with all enriched genes residing in this chromatin. In contrast, the HC dataset shows that the enriched genes come from three different chromatins, with limited existing evidence for them, suggesting that they may arise from noise and are less biologically relevant.

Besides NCBP3, PLAUR—ranked sixth in our analysis—has been reported to have a direct association with HIV. Specifically, PLAUR inhibits HIV-1 virion release from the cell membrane and reduces viral transmission (Pang *et al.* 2023). A detailed analysis of PLAUR is provided in the Supplementary Material, available as supplementary data at *Bioinformatics* online. These findings further validate the effectiveness of scPOEM in integrating scATAC-seq and scRNA-seq data, enabling accurate co-embedding of genes and peaks. Additionally, it offers a new approach to investigate gene regulation by first considering related peaks and then examining the relationship between the target gene and those enriched by these peaks. Moreover, by following a similar scheme, this differential analysis framework can be used to detect regulatory variations between different cell types.

## 4 Conclusion

In this study, we proposed scPOEM, a novel single-cell multi-omics embedding method that integrates scATAC-seq and scRNA-seq data to uncover regulatory relationships between chromatin accessibility peaks and gene expression. By constructing a heterogeneous network that includes peak–peak, peak–gene, and gene–gene relationships, scPOEM generated low-dimensional embeddings that effectively capture the regulatory landscape of genes and peaks. Our experimental results demonstrated that scPOEM outperformed existing methods in identifying super-enhancer-associated peaks, recovering PCHiC interactions, and conducting subgroup and differential regulatory mechanism analyses.

One key strength of scPOEM is its ability to integrate peak–peak, peak–gene, and gene–gene relationships into a unified framework, enabling the discovery of biologically meaningful patterns. For example, by leveraging the embedded features for differential analysis, scPOEM successfully identified differentially regulated genes implicated in HIV and revealed significant differences in peak–gene regulatory interactions between HC and HIV conditions. Another advantage of scPOEM is its ability to co-embed peaks and genes in the same space. This co-embedding enhances the interpretability of regulatory interactions and facilitates downstream analyses by allowing the simultaneous examination of peaks and genes. For instance, our experiments revealed significant relationships between the pathways identified by the associated gene and peak sets, demonstrating that scPOEM effectively projects functionally related elements into nearby locations in the embedding space. This capability is particularly valuable for uncovering cell-type-specific regulatory mechanisms and identifying potential therapeutic targets.

Despite its strengths, scPOEM has several limitations and areas for improvement. First, its computational complexity may increase with larger datasets. We are developing parallel computing strategies to address this challenge. Second, scPOEM is an entirely unsupervised method. Integrating labelled information may further improve the performance of scPOEM. Third, while scPOEM currently focuses on pairwise peak–gene relationships, extending the framework to model peak–gene–protein relationships and co-embed these three entities could enhance its utility further.

## Supplementary Material

btaf483_Supplementary_Data

## Data Availability

All the data used in this article are available from public sources, as detailed above in Section 2.6.
