# Supplementary material for: scPOEM: robust co-embedding of peaks and genes revealing peak–gene regulation
Source: Bioinformatics. 2025 Sep 1;41(9):btaf483. doi: 10.1093/bioinformatics/btaf483 (PMC12449255; doi:10.1093/bioinformatics/btaf483)
Supplement: btaf483_Supplementary_Data [file btaf483_supplementary_data.zip › Supplementary1_scPOEM_final.pdf]

The Supplementary Materials of "scPOEM: Robust Co-embedding of Peaks and Genes Revealing Peak-Gene Regulation"

Yan Zhong[1,†,*], Yuntong Hou[1,†], Yongjian Yang[3], Xinyue Zheng[1] and James J. Cai[2,3,4,*]

1 KLATASDS-MOE, School of Statistics, East China Normal University, Shanghai, China.
2 Department of Veterinary Integrative Biosciences, Texas A&M University, College Station, TX 77843, USA.
3 Department of Electrical and Computer Engineering, Texas A&M University, College Station, TX 77843, USA.
4 Interdisciplinary Program of Genetics, Texas A&M University, College Station, TX 77843, USA.

[†]Yan Zhong and Yuntong Hou contributed equally to this work.
*Corresponding Authors: Yan Zhong, yzhong@fem.ecnu.edu.cn; James J. Cai, jcai@tamu.edu

Details of Methods

Constructing peak-gene networks by Lasso, Random Forest (RF), and XGBoost

For the Lasso method, a linear regression model is constructed via solving a penalized least square optimization problem. Each peak (or predictor) is associated with an estimated coefficient, which will be zero if the peak does not affect the gene expression. Then the (i, j)-th entry of $\boldsymbol{W}^{pg}_{\mathrm{Lasso}}$ is filled by the absolute value of the estimate coefficient of the i-th peak on the j-th gene, or is zero when the i-th peak is not within the 100 kbp upstream and downstream of the gene. The implementation of the Lasso method is by the glmnet (Friedman et al. 2010) R Package, and the tuning parameter in the Lasso method is selected using the Bayesian information criterion (BIC) in practice.

The RF method constructs multiple decision tree models via bootstrapping samples and features and aggregates them together to predict gene expression. We compute the importance score of each peak by its contribution to reducing the mean squared error (MSE) across all decision trees and select a set of peaks with the highest values as the important peaks that potentially affect the gene's expression, whose cumulative importance exceed 90% or individual importance larger than 5% of the total. The (i, j)-th entry of $\boldsymbol{W}^{pg}_{\mathrm{RF}}$ is filled by the importance score of the i-th peak on the j-th gene if the i-th peak is denoted as the important peaks and zero verse vice.

Similar to the RF method, XGBoost also generates a series of decision tree models but via a boosting sequence. We construct the importance score of each peak by assessing improvement in the loss function from splits involving each peak. We select peaks with the highest scores, whose cumulative importance exceed 90% or individual importance larger than 5% of the total. Then we construct $\boldsymbol{W}^{pg}_{\mathrm{XGBoost}}$ in the same way of the RF method.

Details of Performance Evaluation

Super-enhancer detection evaluation

Super enhancers are regulatory regions consisting of multiple enhancers with high transcription factor occupancy, playing a vital role in driving cell-type-specific gene expression. Accurately identifying super enhancers helps reveal key regulatory mechanisms and better understand gene control in different biological contexts. To assess the effectiveness of our method in identifying super-enhancer-associated peaks, we utilized the SEA database (Chen et al. 2020), which contains super-enhancers and their target genes for various cell types. For each paired scRNA-seq and scATAC-seq dataset, we selected the SEA data corresponding to its cell type and retained the enhancer-gene pairs for genes included in our study, which served as the test dataset for peak-gene detection. From the SEA database, we also observe that most super-enhancers locate within the 200 kb of the gene body and therefore focus on peaks in this region of each gene in evaluation.

Two evaluation measures were employed. First, we compared scPOEM with Lasso, Random Forest (RF), and XGBoost regression models in detecting peak-gene pairs. For each method, we extracted the top 3, 5, 7, and 9 peaks within the 200 kb of the gene body for each gene based on their predicted regulatory strength as the predicted meaningful peak-gene pairs. We then calculated the precision and recall of these predicted pairs in recovering the super-enhancer-gene pairs. Higher precision and recall values indicate better performance in peak-gene pair detection. Second, we evaluated the overall distances of peaks for each gene in the latent space using scPOEM. For different regions around genes, we generated boxplots of the distances of peaks located within and outside the super-enhancer regions, binned into 50 kb intervals, respectively. A one-sided Wilcoxon rank-sum test was used to test the null hypothesis that peaks overlapping with super-enhancers are not assigned a higher regulatory strength than non-overlapping peaks.

PCHiC identification evaluation

PCHiC maps physical interactions between gene promoters and distal regulatory elements, providing critical insights into three-dimensional genome organization and cell-type-specific gene regulation. To evaluate the effectiveness of our method in identifying PCHiC interactions, we utilized a publicly available PCHiC dataset (Javierre et al. 2016) as the test dataset, which contains promoter-interaction pairs for genes. These pairs served as the benchmark for assessing the accuracy of our method in detecting promoter-linked regulatory interactions. Specifically, we evaluated the overlapping number between the closest peak-gene pairs by scPOEM and PCHiC interaction regions.

Subgroup regulatory mechanism via enrichment analysis

To evaluate our method's effectiveness in identifying gene groups with regulatory mechanisms, we performed clustering on the embedded features and used gene enrichment analysis to explore the potential regulatory within each cluster. Specifically, we applied K-means algorithm to group genes into clusters of 10~50 genes. For each cluster, we assigned it with peaks that meet two criteria: (1) they are among the top 10 nearest peaks for at least one gene in the cluster, and (2) they are the closest to the cluster center relative to all other clusters.

We first performed gene set enrichment analysis using EnrichR (Kuleshov et al. 2016) with the Gene Ontology (GO) Biological Process library to determine associated biological functions. Then we performed the peak set enrichment analysis via GREAT to determine associated gene and pathways (McLean et al. 2010). All significant pathways from the two enrichments suggest that the peaks and genes in that cluster may be involved in specific regulatory mechanisms. These pathways could highlight potential biological processes where the clustered genes and associated peaks play key roles in regulation.

Differential Analysis for Disease Mechanism Identification

To evaluate scPOEM's ability to identify disease-associated regulatory factors, we compared results between healthy and HIV-infected memory CD4+ T cells. Based on the embedded features, we employed the manifold alignment technique proposed by scTenifoldNet (Osorio et al. 2020) to detect differentially regulated genes between the two conditions. Specifically, for each condition, we focused on the embedded features of genes and constructed a ϵ-nearest neighbor network, which serves as a refined gene regulatory network integrating information from both scATAC-seq and scRNA-seq data. Here, ϵ was set to the 5th percentile of all pairwise gene–gene distances in the embedding space. We then applied the manifold alignment step of scTenifoldNet to align the two networks and detect differentially regulated genes. We then further analyzed the regulations of top differential genes and the peaks closest to it in two conditions respectively.

Basic Statistics and Embedding Results of the Joint Peak-gene Network

Table S1. Basic statistics of the joint peak-gene network constructed by scPOEM for four datasets.

Study	Dataset	Number of peak nodes	Number of gene nodes	Number of peak-peak edges	Number of peak-gene edges	Number of gene-gene edges
1	PBMC	45,764	3,000	878,352	32,369	450,000
2	BMMC	54,212	3,000	1,259,800	37,493	450,000
3	HC	41,497	3,653	702,772	38,015	667,222
	HIV	41,625	3,653	754,190	37,343	667,222

The basic statistics of the joint peak-gene network are shown in Table S1. The detailed embedding values of all nodes (peaks and genes) for the four datasets are available in the fold of "real_data/embedding_results" at https://github.com/Houyt23/scPOEM.

Computing Time for scPOEM

Table S2. Computing time for scPOEM on four datasets using a single core of Intel Xeon Platinum 8260L CPU. This time can be reduced by utilizing multiple cores and parallel computing.

Dataset	Joint Network Construction (hrs)	Meta-path Construction+ Co-Embedding (hrs)	Total (hrs)

PBMC	3.7	3.5	7.2
BMMC	4.9	4.5	9.4
HC	10.7	2.5	13.2
HIV	8.5	2.5	11.0

The Impact of Meta-path Length

To evaluate the impact of meta-path length, we compared five alternative meta-path designs with lengths ranging from 1 to 5:

(a) P1–G1;
(b) P2–P1–G1–G2;
(c) P3–P2–P1–G1–G2–G3;
(d) P4–P3–P2–P1–G1–G2–G3–G4;
(e) P5–P4–P3–P2–P1–G1–G2–G3–G4–G5.

For each meta-path, we sampled the same number of positive node pairs and generated embeddings accordingly. We then assessed their ability to identify super-enhancer–associated peak–gene pairs using the PBMC dataset. As shown in Figure S1, the shortest meta-path (P1–G1), which only considers direct peak–gene links, performed the worst. This confirms that incorporating higher-order relations significantly improves performance.

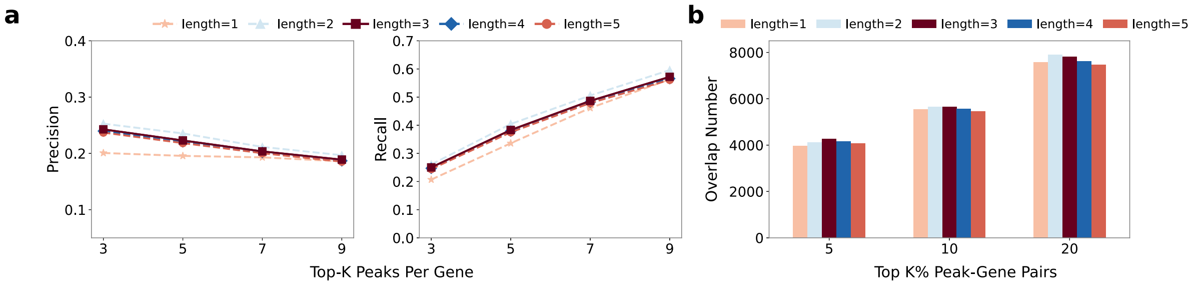

Figure S1. Performance comparison across different meta-path lengths. (a) Precision and recall in identifying super-enhancer–associated peak-gene links. (b) Number of peak-gene links overlapping with Hi-C interactions.

Among the longer meta-paths (lengths 2–5), we observed relatively small performance differences, with lengths 2 and 3 consistently achieving the best results. To further examine the utility of these paths, we analyzed whether the genes enriched by peaks in each meta-path are well covered by the resulting gene subgroups (considering only genes within the retained HVGs). The results, shown in Figure S2, indicate that meta-paths of lengths 3 and 4 provided the highest subgroup coverage, with a median value equal to 1. This suggests that the gene subgroups inferred using these paths are particularly effective in capturing biologically meaningful associations.

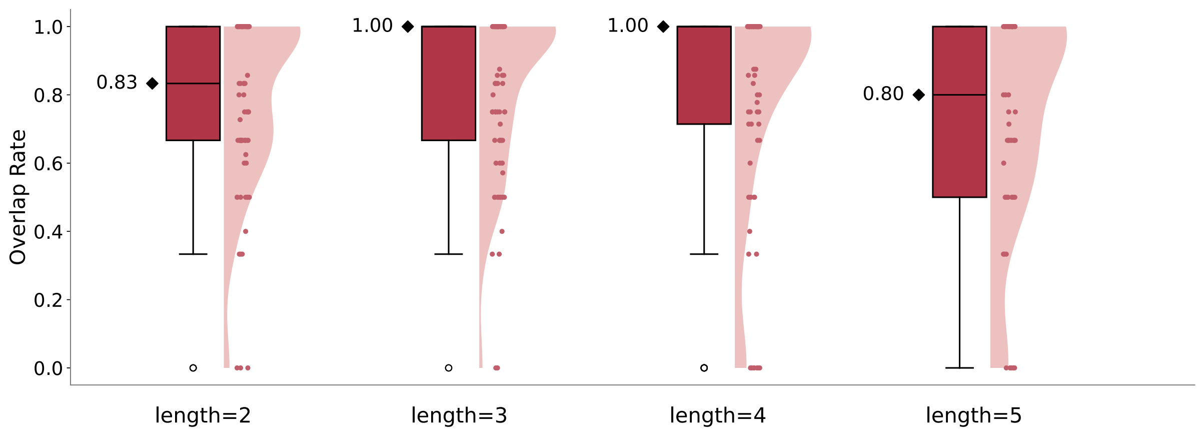

Figure S2. Distribution of the percentage of peak-enriched genes that are covered by the corresponding gene subgroup, considering only genes within the retained HVGs.
Based on these findings, we chose the length-3 meta-path for our main analysis, as it offers good empirical performance while maintaining model interpretability and computational efficiency.

More Results of the Differential Analysis

Except for NCBP3 among the top-ranked genes, we also highlight the role of PLAUR, which ranks sixth in significance in our differential analysis. Previous studies have reported a direct association between PLAUR and HIV, showing that PLAUR inhibits HIV-1 virion release from the cell membrane and reduces viral transmission (Pang et al. 2023). Applying a similar analysis approach as for NCBP3, we observe in Figure S3a that XRCC1—a gene known to be important in HIV infection (Liu et al. 2022)—is specifically enriched by peaks around PLAUR under the HIV condition. Furthermore, functional enrichment analyses shown in Figures S3b–c reveal that peaks surrounding PLAUR in the HIV group are linked to a broader spectrum of biological processes, notably those involved in DNA repair pathways such as single-strand break repair, base-excision repair, and positive regulation of DNA ligation. These pathways have previously been implicated in HIV-related cellular responses (Hrecka et al. 2016).

Together, these findings demonstrate that the regulatory differences we observe are not restricted to NCBP3 but extend to other highly ranked genes like PLAUR, thereby validating the effectiveness of scPOEM.

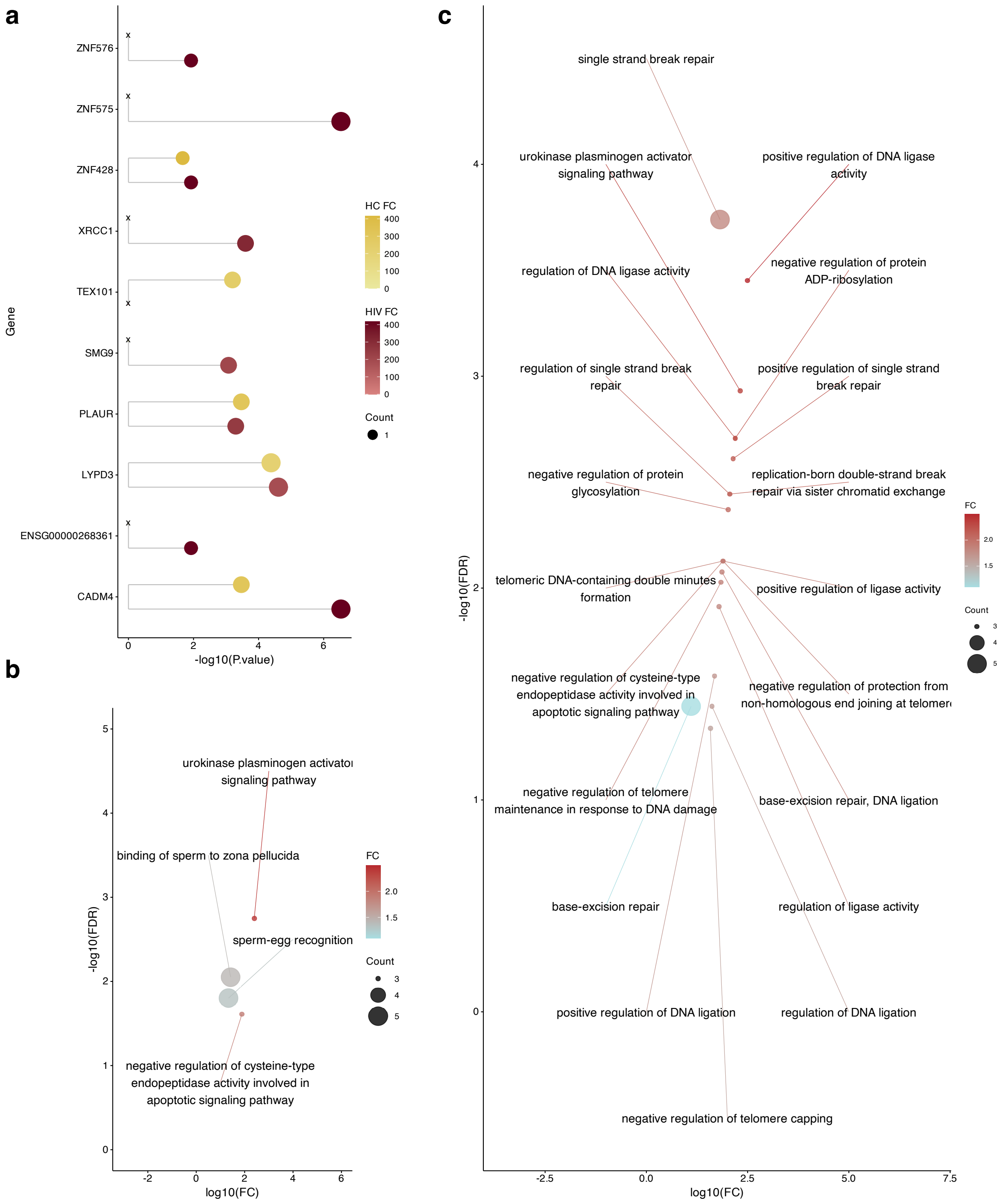

Figure S3. Enrichment analysis of peak sets for PLAUR. (a) Gene enrichment analysis of peaks near PLAUR. (b–c) Pathway enrichment analysis of peaks near PLAUR under HC and HIV conditions, respectively.

Reference

Chen, Chuangeng, Dianshuang Zhou, Yue Gu, Cong Wang, Mengyan Zhang, Xiangyu Lin, Jie Xing, Hongli Wang, and Yan Zhang. "SEA version 3.0: a comprehensive extension and update of the Super-Enhancer archive." Nucleic acids research 48, no. D1 (2020): D198-D203.

Friedman, Jerome H., Trevor Hastie, and Rob Tibshirani. "Regularization paths for generalized linear models via coordinate descent." Journal of statistical software 33 (2010): 1-22.

Hrecka, Kasia, Caili Hao, Ming-Chieh Shun, Sarabpreet Kaur, Selene K. Swanson, Laurence Florens, Michael P. Washburn, and Jacek Skowronski. "HIV-1 and HIV-2 exhibit divergent interactions with HLTF and UNG2 DNA repair proteins." Proceedings of the National Academy of Sciences 113, no. 27 (2016): E3921-E3930.

Javierre, Biola M., Oliver S. Burren, Steven P. Wilder, Roman Kreuzhuber, Steven M. Hill, Sven Sewitz, Jonathan Cairns, Steven W. Wingett, Csilla Várnai, Michiel J. Thiecke, Frances Burden, Samantha Farrow, Antony J. Cutler, Karola Rehnström, Kate Downes, Luigi Grassi, Myrto Kostadima, Paula Freire-Pritchett, Fan Wang, The BLUEPRINT Consortium, Hendrik G. Stunnenberg, John A. Todd, Daniel R. Zerbino, Oliver Stegle, Willem H. Ouwehand, Mattia Frontini, Chris Wallace, Mikhail Spivakov, and Peter Fraser. "Lineage-specific genome architecture links enhancers and non-coding disease variants to target gene promoters." Cell 167, no. 5 (2016): 1369-1384.

Kuleshov, Maxim V., Matthew R. Jones, Andrew D. Rouillard, Nicolas F. Fernandez, Qiaonan Duan, Zichen Wang, Simon Koplev, Sherry L. Jenkins, Kathleen M. Jagodnik, Alexander Lachmann, Michael G. McDermott, Caroline D. Monteiro, Gregory W. Gundersen, and Avi Ma'ayan. "Enrichr: a comprehensive gene set enrichment analysis web server 2016 update." Nucleic acids research 44, no. W1 (2016): W90-W97.

Liu, Bangquan, Kaili Wang, Jiawei Wu, Yuanting Hu, Xun Yang, Lidan Xu, Wenjing Sun et al. "Association of APEX1 and XRCC1 gene polymorphisms with HIV-1 infection susceptibility and AIDS progression in a northern Chinese MSM population." Frontiers in Genetics 13 (2022): 861355.

McLean, Cory Y., Dave Bristor, Michael Hiller, Shoa L. Clarke, Bruce T. Schaar, Craig B. Lowe, Aaron M. Wenger, and Gill Bejerano. "GREAT improves functional interpretation of cis-regulatory regions." Nature biotechnology 28, no. 5 (2010): 495-501.

Osorio, Daniel, Yan Zhong, Guanxun Li, Jianhua Z. Huang, and James J. Cai. "scTenifoldNet: a machine learning workflow for constructing and comparing transcriptome-wide gene regulatory networks from single-cell data." Patterns 1, no. 9 (2020).

Pang, Hailin, Jiayue Ouyang, Zengwen Yang, Hong Shang, and Guoxin Liang. "Urokinase plasminogen activator surface receptor restricts HIV-1 replication by blocking virion release from the cell membrane." Proceedings of the National Academy of Sciences 120, no. 3 (2023): e2212991120.
